# The role of oncology nurse navigators in the outcomes of surgical patients with head and neck cancer

**DOI:** 10.1590/0034-7167-2024-0165

**Published:** 2025-12-08

**Authors:** Debora Costa Miguel Gobo, Rossana Veronica Mendoza Lopez, Adriana Marques Silva, Marco Vamondes Kulcsar

**Affiliations:** I Universidade de São Paulo. São Paulo, São Paulo, Brazil; II Centro de Investigação Translacional em Oncologia, Instituto do Câncer do Estado de São Paulo. São Paulo, São Paulo, Brazil; III Instituto do Câncer do Estado de São Paulo. São Paulo, São Paulo, Brazil

**Keywords:** Patient Navigation, Head and Neck Neoplasms, Surgical Oncology, Outcome Assessment, Health Care, Nurses., Navegación de Pacientes, Neoplasias de Cabeza y Cuello, Oncología Quirúrgica, Evaluación de Resultado en la Atención de Salud, Enfermeras y Enfermeros.

## Abstract

**Objectives:**

to assess oncology nurse navigators’ performance in surgical patients with head and neck cancer.

**Methods:**

this observational, retrospective cohort study analyzed oncology nurse navigators’ performance, comparing the intervention group (monitored by a nurse navigator) and control group (institutional flow).

**Results:**

a total of 160 patients were included: 40 in the intervention group and 120 in the control group. The intervention group showed a 28-day reduction in speech-language pathology visits, 26 in psychology visits, 31 in social services visits, and ten in CT scan visits. The intervention group also saw a 53% reduction in emergency room visits and a 20% reduction in “Hello, Nurse” calls.

**Conclusions:**

oncology nurse navigators’ performance contributed to reducing the time for surgery, staging tests, care with a multidisciplinary team, better adherence to treatment, reduction of “Hello, Nurse” calls and visits to the emergency room, thus confirming oncology nurse navigators’ relevance in the oncology care center.

## RESUMEN

**Objetivos:**

evaluar el desempeño de las enfermeras navegadoras en pacientes quirúrgicos con cáncer de cabeza y cuello.

**Métodos:**

estudio de cohorte observacional y retrospectivo analizó el desempeño de las enfermeras navegadoras, comparando el grupo de intervención (monitoreado por una enfermera navegadora) con el grupo control (flujo institucional).

**Resultados:**

se incluyeron 160 pacientes: 40 en el grupo de intervención y 120 en el grupo control. El grupo de intervención mostró una reducción de 28 días en las visitas a logopedia, 26 en las visitas a psicología, 31 en las visitas a servicios sociales y 10 en las visitas a tomografías computarizadas. El grupo de intervención también observó una reducción del 53% en las visitas a urgencias y del 20% en las llamadas de “Hola, Enfermera”.

**Conclusiones:**

la actuación del enfermero navegador contribuyó a la reducción del tiempo de cirugía, de exámenes de estadificación, de atención con equipo multidisciplinario, mejor adhesión al tratamiento, reducción de llamadas al “Hola, Enfermera” y de visitas a urgencias, confirmando así la relevancia del enfermero navegador en el centro de atención oncológica.

## INTRODUCTION

Advanced head and neck cancer (HNC) is multifactorial, and its treatment is multimodal, involving different therapeutic modalities, such as radiotherapy, chemotherapy, and surgery, either alone or in combination. Approximately 70 to 80% of HNC patients present with advanced disease at diagnosis and risk factors associated with chronic tobacco and alcohol consumption as well as nutritional and cardiorespiratory disorders^(1)^.

The complexity and severity of this disease reinforce the importance of close monitoring by nurses involved in care. In this context, oncology nurse navigators (ONNs) aim to facilitate the diagnosis and ensure continuity of treatment for cancer patients. These professionals work with patients and their families, helping them overcome the socioeconomic, cultural, bureaucratic, and psychological barriers that hinder access to healthcare services and systems, in addition to optimizing adherence and contributing to treatment effectiveness^(2)^.

Studies assessing ONNs’ performance showed that lung cancer patients monitored by ONNs started their treatment earlier than patients not monitored by the same group^(3,4)^. Another study found a reduction in the time between breast cancer diagnosis and the start of treatment^(5)^. Both publications highlight the relevance of ONNs’ work.

Given this evidence, navigation programs for cancer patients are gradually being implemented in Brazil at various high-complexity oncology centers. Despite this, there are still few studies demonstrating the effectiveness of ONN, especially those focused on surgical patients with HNC.

Thus, considering the complexity of caring for patients with HNC, it becomes relevant to study the role of ONNs in navigation during the surgical treatment of these individuals.

## OBJECTIVES

To assess the impact of ONNs’ performance in the treatment of surgical patients with HNC.

## METHODS

### Ethical aspects

The research was approved by the Research Ethics Committee in accordance with Brazilian Resolution 466/2012 for research involving human subjects. Informed Consent Forms (ICFs) were provided to all available patients who presented to the service. In cases of death, ICF requirement waiver was requested. In cases of inability to attend the institution due to the COVID-19 pandemic, the need for social isolation, or other limiting conditions, consent was provided over the phone using Iris Dialtech^®^ (standardized at the institution). The calls were recorded, ensuring that patient understood the confidentiality guarantee and agreed to the use of their data. This article is the result of a master’s thesis entitled “*Atuação do enfermeiro navegador no desfecho dos cuidados pré e pós-operatórios em pacientes com tumor de cabeça e pescoço no Instituto do Câncer do Estado de São Paulo*”. The dissertation in question is available in the *Universidade de São Paulo*’s Digital Library of Theses and Dissertations, and can be accessed through the following link: https://doi.org/10.11606/D.5.2024.tde-12092024-163018.

### Study design, period and location

This is an observational, retrospective, quantitative cohort study carried out at a High Complexity Oncology Care Center, which receives and treats patients with HNC from all regions of Brazil and Latin America, regulated by the Brazilian Health System, from December 2018 to March 2021.

At this study site, there is a program for assisting patients with HNC by an ONN, which takes place in a systematic manner as follows:

Patients arrive at our clinic already diagnosed with HNC. Upon their first consultation with the head and neck surgery team, those classified as stage >II are referred for assessment by an ONN (National Registered Nurse), who, through a nursing consultation, assesses patients’ health needs (e.g., social, nutritional, economic, psychological, and other issues), and then refers patients for care by the multidisciplinary team.

ONNs monitor and manage consultations, assess reasons for missed consultations, and actively search for patients when necessary to facilitate patients’ journey and processes during treatment. Furthermore, ONNs discuss the case during clinical meetings, which involve the participation of interdisciplinary teams, including the surgical, oncology, radiation therapy, and radiology teams. This meeting (called a Tumor Board) analyzes the staging tests, addressing patients’ social, nutritional, and psychological issues that could impact treatment. This clinical discussion determines whether a patient will continue with the surgical schedule or undergo a change in clinical treatment. The cancer center offers a 24-hour telephone support program for patients, family members, and caregivers called “Hello, Nurse” (*Alô Enfermeiro*). Patients and their family members/caregivers can have their questions regarding treatment and/or procedures answered from home, providing greater convenience and safety.

STrengthening the Reporting of Observational Studies in Epidemiology recommendations guided the study design.

### Population or sample; inclusion and exclusion criteria

Patients diagnosed with HNC after their first medical consultation at the institution, with stages II to IV of the disease, who had indications for major surgery and who had indications for tracheostomy and nasogastric tube use, were included. Patients with malignant tumors of the thyroid gland, salivary glands, or skin were excluded.

### Study protocol

The study was divided into two phases:

- 1^st^ phase (staging): this phase consisted of HNC patients assisted from the first institutional consultation (FIC) (moment of patient admission to the hospital) until the definition of conduct, after staging tests and discussion at the Tumor Board/medical follow-up;
- 2^nd^ phase (operative): this phase was composed after the management decision was made by the Tumor Board/medical follow-up until postoperative follow-up. In this phase, patients whose management consisted solely of clinical follow-up were excluded.

The sample size calculation was based on the comparison of ONNs’ performance (intervention group - patients who were followed by an ONN), considering a type I error of 5% and statistical power of 80%, with the hypothesis that 50% of the intervention group would have the best treatment follow-up and the null hypothesis that 30% of the control group (not followed by an ONN, followed the institutional flow) would have better treatment follow-up.

The control and intervention groups were each divided into two subgroups. Thus, we had:

- Intervention and surgery group: patients who were monitored by an ONN and underwent surgery;
- Intervention and clinical treatment group: patients who were monitored by an ONN and clinical treatment was indicated;
- Control and surgery group: patients who were not monitored by an ONN and underwent surgery;
- Control and clinical treatment group: patients who were not monitored by an ONN and were prescribed clinical treatment.

**Figure 1:**
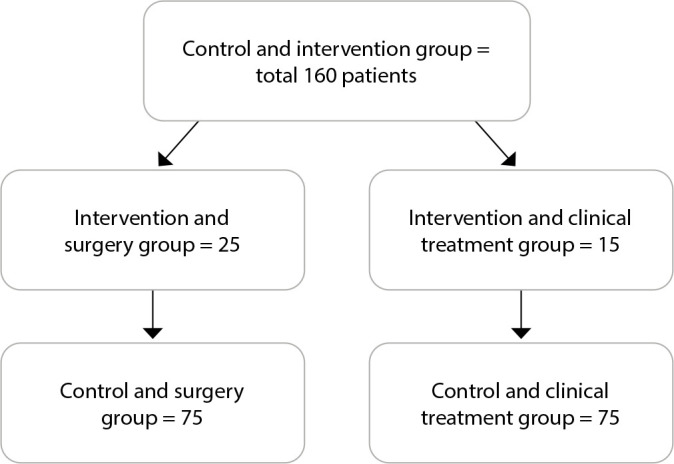
Description of groups

The number of cases was 40 patients in the intervention group, and after the 1^st^ phase, 25 were referred for surgery (intervention and surgery group) and 15 were referred for clinical conduct (intervention and clinical treatment group). In the control group, of the 120, 75 patients were operated on (control and surgery group) and 45 were referred for clinical conduct (control and clinical treatment group), being considered 3:1, as shown in Figure 1.

Quantitative data collection was performed through a retrospective search of electronic medical records for patients who participated in ONN follow-up (intervention and surgery group and intervention and clinical treatment group). These data were compared with patients who attended the outpatient clinic during the same period and had the same characteristics (ICD 10 - International Classification of Diseases - disease staging). A separate spreadsheet was created for patients who underwent surgery and those who received clinical treatment. This spreadsheet was entered into the Randon System, and patients were randomized until the number of patients for the control group (control and surgery group) and (control and clinical treatment group) was reached.

To support data collection, a data collection instrument and a database were developed on the REDCap^®^ platform to record information collected from electronic medical records. All collected data were confidential, with access only by the investigator, ensuring data confidentiality.

To assess the performance and benefits of ONN care for patients with HNC, the following variables were assessed and compared between the intervention and control groups: level of education; clinical performance assessment using the Eastern Cooperative Oncologic Group score, Body Mass Index, and Human Development Index (HDI); time between PCI and staging tests; time elapsed from diagnosis to start of treatment; time between the first nutritional consultation and consultation with an interdisciplinary team (nutrition, speech therapy, social work, psychology, and dentistry); length of hospital stay; number of patients who sought emergency care; number of patients who contacted the “Hello, Nurse” service.

### Analysis of results and statistics

Qualitative variables were presented as absolute (n) and relative (%) frequencies, and quantitative variables were presented as mean, median, minimum and maximum values, and standard deviation. Associations between qualitative variables were analyzed using Pearson’s chi-square test or Fisher’s exact test. Comparisons between quantitative variables between groups were performed using Student’s t-test or Mann-Whitney test, depending on data distribution. The significance level was set at 5%. Statistical analyses were performed using the Statistical Package for the Social Sciences version 18 for Windows.

## RESULTS

After meeting the eligibility criteria, a total of 160 patients were included in the study, of which 40 comprised the intervention group, and 120 were included in the control group.

In the population that comprised the study, age ranged from 30 to 85 years among men and women, with a median of 60.3 in the intervention group and 53.5 in the control group.

As for sex, both in the intervention and control groups, there was a predominance of males, as well as a higher frequency of incomplete elementary education, high HDI in both groups and T4 staging.

Concerning diagnosis, tongue neoplasia was the most prevalent in both the intervention group and the control group, with no statistically significant difference between groups.

A survey of the time interval between the date of patients’ FIC and the date they were seen by the multidisciplinary team was conducted. For the speech-language pathology, psychology, and social work teams, the intervention group received care in approximately two days, two days, and 1.37 days, respectively. This accounts for a reduction of approximately 93% compared to the control group, which waited 30 days, 25.6 days, and 33.64 days, respectively. For CT scans, the intervention group waited an average of 9.24 days, while the control group waited 19.77 days. This accounts for a reduction of approximately 53% in waiting time (from 19.77 days to 9.24 days). The difference was statistically significant (p = 0.01). However, it was observed that the intervention group took, on average, longer to undergo the dental assessment compared to the control group (Table 1).

**Table 1:** Description of the intervention and control groups according to the time interval between the first institutional consultation with the interdisciplinary team and staging tests (tomography and endoscopy - *Instituto do Câncer do Estado de São Paulo - Hospital das Clínicas* **, School of Medicine,** *Universidade de São Paulo* - 2018 to 2021), São Paulo, São Paulo, Brazil, 2024

	Control group		Intervention group		*p* value
	Mean	Standard deviation	Mean	Standard deviation	
Between FIC and nursing consultation	15.89	69.66	0.11	0.46	0.004^3^
Between FIC and nutrition consultation	18.71	29.77	1.68	0.67	0.113^3^
Between FIC and speech therapy consultation	30	24.97	2	<0.01	**0.000^3^**
Between FIC and psychology consultation	25.6	31.96	2	<0.01	**0.000^3^**
Between FIC and social service consultation	33.64	33.61	1.37	0.96	**0.000^3^**
Between FIC and dentist consultation	33.23	34.14	44.28	172.66	**0.000^3^**
Between FIC and CT scan	17.56	23.03	6.72	5.9	**0.001^3^**
Between FIC and endoscopy	19.77	23.94	9.24	5.48	0.321^3^

3 Mann-Whitney test; FIC - first institutional consultation.

In relation to adherence to treatment, it was found that the intervention group presented better adherence to treatment when compared to the control group, presenting a statistically significant association (p<0.001) (Table 2)

**Table 2:** Description of the intervention and control groups according to assessments regarding patient adherence to consultations (*Instituto do Câncer do Estado de São Paulo - Hospital das Clínicas* **, School of Medicine,** *Universidade de São Paulo* - 2018 to 2021), São Paulo, São Paulo, Brazil, 2024

	Control group		Intervention group		*p* value
	n	%	n	%	
Yes	51	42.5	30	75	**<0.001^2^**
No	69	57.5	10	25	
Absences	69	57.5	10	25	
No companion^*^	NA	NA	2	5	
No financial means^*^	NA	NA	3	7.5	
Forgetfulness^*^	NA	NA	<0.01	<0.01	

^2^Fisher’s exact test;

* Intervention group and control group; NA - not applicable.

In relation to the time between the first consultation and surgery, the mean number of days was shorter in the intervention group. The length of hospital stay (between surgery and discharge) was longer in the intervention group, although there was no statistically significant association between the groups. This result can be seen in Table 3.

**Table 3:** Description of the intervention and surgery group and the control and surgery group according to the time interval between the first institutional consultation and surgery, and surgery and discharge (*Instituto do Câncer do Estado de São Paulo - Hospital das Clínicas* **, School of Medicine,** *Universidade de São Paulo* - 2018 to 2021), São Paulo, São Paulo, Brazil, 2024

		Control and surgery group	Intervention and surgery group	*p* value
**Time between 1^st^ consultation and surgery (days)**	Mean	65.75	41.92	0.006^3^
	Median	54	34	
	Minimum	13	19	
	Maximum	301	89	
**Time from surgery to discharge (days)**	Mean	17.39	28.48	0.4393^3^
	Median	8	10	
	Minimum	0	2	
	Maximum	360	356	

*^3^Mann-Whitney test.*

Regarding the demand for the “Hello, Nurse” service, it was found that it was higher in the control and surgery groups compared to the other groups.

Questions about nasogastric tube and tracheostomy care were the most frequent among patients in the control and intervention groups undergoing surgery. Among those undergoing clinical treatment, the main complaint was unmitigated pain. However, there was no statistically significant difference between the groups (Table 4).

**Table 4:** Description of the intervention (clinical treatment and surgery) and control (clinical treatment and surgery) groups according to the search for “Hello, Nurse” and the reasons for the call, São Paulo, São Paulo, Brazil, 2024

	Control and surgery group		Intervention and surgery group		*p* value	Control group and clinical treatment		Clinical intervention and treatment group		*p* value
	**n**	**%**	**n**	**%**		**n**	**%**	**n**	**%**	
**“Hello Nurse” call**					0.107^2^					0.541^2^
Yes	42	56.00	9	36.00		17	37.80	4	26.70	
No	33	44.00	16	64.00		28	62.30	11	73.30	
**Reason**										
Questions related to care (nasoenteral catheter/tracheostomy)	16	38.10	2	22.20		1	5.90	<0.01	<0.01	
Pain	3	7.10	<0.01	<0.01		7	41.20	1	25.00	
Clinical complaints (hypotension/malaise/surgery wound secretion/fever)	16	38.10	2	22.20		4	23.50	<0.01	<0.01	
Questions about scheduling	11	26.20	4	44.40		3	17.60	3	75.00	
Contact with navigator	<0.01	<0.01	1	11.10		<0.01	<0.01	<0.01	<0.01	
Other	5	11.90	2	22.20		1	5.90	<0.01	<0.01	

2 Fisher’s exact test. The follow-up time and calls to “Hello, Nurse” were from the first institutional consultation to the postoperative consultation for the intervention/control and surgery groups, and from the first institutional consultation to before starting clinical treatment for the intervention/control and clinical treatment groups.

Concerning emergency room visits, it was observed that the demand was higher in the control and surgery groups, as well as in the control and clinical treatment groups. The most frequent complaint in both groups was untreated pain, with a statistically significant difference of p<0.001 and p=0.003, respectively, as shown in Table 5.

**Table 5:** Description of the intervention (clinical treatment and surgery) and control (clinical treatment and surgery) groups according to emergency room attendance and reason, São Paulo, São Paulo, Brazil, 2024

	Control and surgery group		Intervention and surgery group		*p* value	Control group and clinical treatment		Clinical intervention and treatment group		*p* value
	**n**	**%**	**n**	**%**		**n**	**%**	**n**	**%**	
**Coming to the emergency room**					0.000^2^					0.003^2^
Yes	61	81.3	7	28.0		35	77.8	5	33.3	
No	14	18.7	18	72.0		10	22.2	10	66.7	
**Reason**										
Pain	24	40.0	<0.01	<0.01		10	28.6	2	40.0	
Bleeding	3	5.6	<0.01	<0.01		2	5.7	1	20.0	
Dyspnea	2	3.1	1	14.2		6	17.1	1	20.0	
Nasogastric catheter loss	14	23.3	1	14.2		3	8.6	1	20.0	
Tracheostomy-related	1	1.7	<0.01	<0.01		7	20.4	<0.01	<0.01	
Other infections	7	11.7	2	28.5		<0.01	<0.01	<0.01	<0.01	
Surgical wound infections	6	8.1	1	14.2		4	11.4	<0.01	<0.01	
Drain loss	1	1.7	<0.01	<0.01		<0.01	<0.01	<0.01	<0.01	
Other	3	5.6	2	28.5		3	8.6	<0.01	<0.01	

2 Fisher’s exact test. The follow-up time and calls to “Hello, Nurse” were from the first institutional consultation to the postoperative consultation for the intervention/control and surgery groups, and from the first institutional consultation to before starting clinical treatment for the intervention/control and clinical treatment groups.

## DISCUSSION

This study indicated that ONN demonstrated an impact on the perioperative care of patients with HNC. The advantage of this study is the simultaneous analysis of two groups of patients with HNC treated at a tertiary oncology center. Another strength is the ONN approach, as this professional integrated and coordinated a multidisciplinary team (nutrition, speech therapy, social work, psychology, and dentistry).

The profile of the HNC patients studied was predominantly male, 94 (78.3%) in the control groups and 35 (87.5%) in the intervention groups. The educational level of the study population was 75 (62.5%) in the control groups and 27 (67.5%) in the intervention groups with incomplete elementary education. Males, as well as low educational level, is predominant in several articles when discussing HNC^(6-9)^.

Advanced T4 staging was predominant in both groups, indicating that patients arrived at the institution with locally advanced tumors, which, in terms of treatment, means a more aggressive, costly approach, and a lower quality of life. Tumors staged T4 are associated with reduced survival, particularly in HNC^(10,11)^. Due to the higher incidence of patients diagnosed at stage 4, it is crucial that these individuals receive close and ongoing monitoring. This ensures that treatment is initiated as quickly as possible, maximizing the chances of efficacy and improving clinical outcomes.

Aiming at the need for early treatment initiation, this study raised time variables, which are already confirmed by previous evidence in the literature, that ONNs optimizes the initiation of treatment in cancer patients^(5,12-15)^. This study compared the time spent with the interdisciplinary team and staging tests between the groups. The mean time spent with speech-language pathology teams in the intervention group was two days, and in the control group (a gain of 28 days), 30 days; for psychology, two days for the intervention group and 28.5 days for the control group (a decrease of 26 days); and for social work, 1.37 days in the intervention group and 33.5 days in the control group (a decrease of 31 days). CT scans were performed on average after 6.72 days in the intervention group and 17.5 days in the control group (ten days less), showing a statistically significant association (p<0.001) in the study population.

Another relevant finding is related to adherence to cancer treatment. According to the World Health Organization, adherence is a multidimensional phenomenon influenced by five sets of factors: socioeconomic (poverty, illiteracy, etc.); health team and system-related (underdeveloped healthcare services, short consultations, etc.); condition-related (symptoms related to disease progression, availability of effective treatments); therapy-related; and patient-related (forgetfulness, psychosocial stress, anxiety, among others)^(16)^.

Thus, patient adherence is crucial to treatment success, proving challenging, as it involves behavioral and subjective aspects. The method chosen in this study to measure adherence was data collection through records of medical consultations, interdisciplinary team visits, and tests. Patients who had two or more unexcused absences from consultations or tests were considered to have poor adherence.

Based on this definition, the present study found that in the intervention group monitored by the ONN, adherence was observed in 30 (75%) of scheduled consultations and tests, compared with 51 (42.5%) in the control group (p<0.01). Studies show that patients monitored by the ONN have better adherence to treatment. This is due to factors such as improved patient satisfaction, which reflects greater adherence^(17-19)^.

Another important point is the bond created between the patient and the ONN. When providing care, it is important to provide a welcoming environment, showing respect and empathy at that moment, in addition to cultural adaptation, respecting patients’ beliefs and values through effective communication, through which a bond of trust is created between the patient and the nurse. This bond is strengthened with each consultation and call, allowing patients to feel more comfortable discussing their difficulties, including whether there will be any impediments to their attendance due to lack of financial resources, for example. This provides an opportunity to contact social services and thus avoid patients’ missed consultations, thus highlighting ONNs’ interface with social services.

Specifically, regarding the influence of ONNs on the intervention and surgical control groups, it is important to highlight that the time between the first consultation and surgery was shorter for the intervention and surgery group, with a median of 34 days and 54 days for the control and surgery groups. The surgical schedule remained unchanged, with no changes in the sequence of operations. Patients in the intervention and surgery groups underwent surgery as scheduled, with no changes in the order of priority; this discrepancy in time may be attributed to improved treatment adherence among patients in the intervention group.

When patients missed tests or consultations in these groups, ONNs conducted an active search to locate these patients, rescheduling tests/consultations, understanding the reason for the absence, and creating an action plan to assist them. As a result, they were ready for surgery sooner, contributing to the observed difference. It is worth noting that this type of patient requires closer monitoring for all the reasons mentioned above.

In relation to length of hospital stay from surgery to discharge, there was no statistical significance between the intervention and control groups, but the median was ten days for the intervention and surgery group and eight days for the control and surgery group. A different result was found in the literature, with a study investigating the effectiveness of systematically planned interventions by ONN in patients recently diagnosed with cancer in general. The results indicated that patients who received ONN care had a shorter hospital stay compared to the control and surgery group^(19-21)^.

Despite being a small difference in the result (two days), a possible justification for the extension of time in the intervention and surgery group was that some patients in the intervention group had low family support, requiring greater social support for discharge planning, which increased the length of hospital stay.

Even considering the interdisciplinary team’s expertise, some challenges arose in the postoperative period, as these patients needed to be referred to a support institution capable of providing comprehensive care. However, obtaining a bed in this type of institution was only possible when the patient was fit for discharge and a bed was available. This waiting period, on some occasions, resulted in considerable delays, contributing to an increase in the median length of hospital stay (days). Therefore, the need to improve the referral and counter-referral system of the Brazilian Health System was identified, recognizing the importance of a coordinated effort to optimize the transfer of patients with specific needs, such as patients with HNC in postoperative recovery.

The literature clearly highlights that the performance of ONN has a positive impact on reducing emergency room visits^(21,22)^. When analyzing the incidence of patients who sought emergency care in this study, it was observed that 61 (81.3%) in the control and surgery group who underwent surgery required emergency room visits, compared to seven (28%) in the intervention and surgery group. In the case of clinical treatment, 35 (77.8%) in the control and clinical treatment group sought emergency care, compared to five (33.3%) in the intervention and clinical treatment group (p<0.01).

An analysis of the reasons these patients sought emergency care revealed pain as the main cause in all study groups, even after frequent patient guidance on proper medication use. During ONN consultations, as well as in discussions with the medical team, pain and the need for medication adjustments were always discussed.

One of the navigation strategies is telenursing, defined by the International Council of Nurses as the practice of remote nursing via electronic means that has shown significant benefits, being recognized as a tool for the management of toxicities and symptoms in cancer patients^(23)^.

At the aforementioned cancer center, *Instituto do Câncer do Estado de São Paulo*, there is a program called “Hello, Nurse”, which involves nurses providing telephone support and monitoring to patients undergoing oncological, surgical, and clinical treatment. The number of daily consultations has steadily increased over the years at the institution. In 2021, the program provided more than 54,000 inbound consultations, in which patients or companions contact the program’s central office, with approximately 30,000 active consultations, in which nurses contact patients or companions^(23)^.

The present study assessed calls from patients monitored by ONNs to the “Hello, Nurse” service. The results obtained show 42 (56.0%) from the control and surgery group and 17 (37.8%) from the control and clinical treatment group, compared with nine (36.0%) from the intervention and surgery group and four (26.7%) from the intervention and clinical treatment group. The main reasons for patient/family calls were questions related to care, such as nasogastric tube care and tracheostomy in both groups. Also noteworthy is the higher number of calls (7; 41.2%) in the control and clinical treatment groups due to complaints of untreated pain, thus raising the need for better analgesia coverage in HNC patients undergoing clinical treatment. The role of ONNs has been widely recognized as an effective strategy for reducing the need for medical care, including emergency room visits and telephone calls^(19,20,22)^.

The role of ONNs is to facilitate actions for patients and families throughout the cancer treatment process. Furthermore, they contribute to reducing the time required for procedures, reducing emergency room visits, better clarifying questions that arise at each stage of treatment, and providing a safe haven for patients and their families, knowing they have a trusted professional who helps them coordinate everything that needs to be done at this stage of their lives.

Regarding the management aspect, the effectiveness of ONN’s actions is reflected in the possibility of developing indicators, such as reducing the time to perform staging tests, assessments by the multidisciplinary team, time from FIC to surgery, length of hospital stay, in addition to monitoring adherence to treatment, calling “Hello, Nurse” and visits to the emergency room.

### Study limitations

As a limitation of this research, we highlight the decrease in the number of patients treated by ONNs during the COVID-19 pandemic, since patients were not referred for FIC due to health restrictions; therefore, there is a reduced number of patients in the intervention group.

### Contributions to health, nursing, or public policy

This study contributes significantly to nursing by highlighting the benefits of ONN’s work, as well as showing positive results in indicators of time, adherence, and reduced emergency room visits. When considering the reduction in emergency room visits, the associated cost reduction is considered. Although this study did not directly address cost reduction, future studies may address this topic.

## CONCLUSIONS

The results of this study allowed us to assess the positive healthcare impact of implementing the ONN for both patients with HNC and the institution.

When comparing the intervention group with the control group, it was concluded that, for surgical patients with HNC, ONNs improved treatment adherence, reduced the time for tests and consultations with a multidisciplinary team, and reduced the median time to surgery by 20 days. It was also observed that patients guided by ONNs had fewer emergency room visits and fewer calls to the “Hello, Nurse” service.

Thus, this study confirmed that the role of ONNs as care coordinators, acting as an interface with the interdisciplinary team, provides more effective communication, acting as a link between the care team, in order to meet the biopsychosocial health needs presented by patients with HNC.

Finally, ONNs place patients at the center of care and thus become a reference both for patients and their families, in the difficult journey of cancer treatment, and for the care team, in coordinating the care actions necessary for each patient, as well as for the institution, by contributing to the identification of early signs of complications and reducing hospitalization days.

## Data Availability

The research data are available only upon request.
