# Supplementary material for: The role of oncology nurse navigators in the outcomes of surgical patients with head and neck cancer
Source: Rev Bras Enferm. 2025 Dec 8;78(Suppl 3):e20240165. doi: 10.1590/0034-7167-2024-0165 (PMC12707756; doi:10.1590/0034-7167-2024-0165)
Supplement: Translation

# Translation: Atuação do enfermeiro navegador no desfecho de pacientes cirúrgicos com câncer de cabeça e pescoço

**DOI:** 10.1590/0034-7167-2024-0165pt

**Authors:** Debora Costa Miguel Gobo, Rossana Veronica Mendoza Lopez, Adriana Marques Silva, Marco Vamondes Kulcsar

**Affiliations:** I Universidade de São Paulo. São Paulo, São Paulo, Brasil; II Centro de Investigação Translacional em Oncologia, Instituto do Câncer do Estado de São Paulo. São Paulo, São Paulo, Brasil; III Instituto do Câncer do Estado de São Paulo. São Paulo, São Paulo, Brasil

**Keywords:** Navegação de Pacientes, Neoplasias de Cabeça e Pescoço, Oncologia Cirúrgica, Medidas de Desfechos, Enfermeiros.

## Abstract

**Objetivos:**

avaliar a atuação do enfermeiro navegador em pacientes cirúrgicos com câncer de cabeça e pescoço.

**Métodos:**

estudo observacional tipo coorte, retrospectivo, que analisou atuação do enfermeiro navegador comparando grupo intervenção (acompanhado pelo enfermeiro navegador) e grupo controle (fluxo institucional).

**Resultados:**

incluíram-se 160 pacientes: 40 compuseram o grupo intervenção e 120, o grupo controle. Observou-se no grupo intervenção uma redução de 28 dias no atendimento da fonoaudiologia, 26, da psicologia, 31, do serviço social e dez, da tomografia, bem como uma redução de 53% de idas ao pronto atendimento e 20% de ligações ao “Alô, Enfermeiro’ pelo grupo intervenção.

**Conclusões:**

a atuação do enfermeiro navegador contribuiu para a redução de tempo para cirurgia, exames de estadiamento, atendimento com equipe multidisciplinar, melhor adesão ao tratamento, redução de ligações no “Alô, Enfermeiro” e de idas ao pronto socorro, confirmando, assim, a relevância do enfermeiro navegador no centro de atendimento oncológico.

## INTRODUÇÃO

O câncer de cabeça e pescoço (CCP) avançado é multifatorial, e seu tratamento, multimodal, sendo realizado por meio de diferentes modalidades terapêuticas, como radioterapia, quimioterapia e cirurgia, de maneira exclusiva ou combinadas. Cerca de 70 a 80% dos pacientes com CCP apresentam doença avançada ao diagnóstico e com fatores de risco associados aos hábitos de consumo crônico de tabaco e bebidas alcoólicas, além de distúrbios nutricionais e cardiorrespiratórios^(1)^.

A complexidade e a gravidade desta doença reforçam a relevância do acompanhamento mais atento por parte dos enfermeiros envolvidos na assistência. Neste contexto, o enfermeiro navegador oncológico (em inglês, *oncology nurse navigator* (ONN)) visa ser um facilitador, desde a confirmação do diagnóstico, e garantir a continuidade do tratamento dos pacientes oncológicos. Esse profissional atua junto aos pacientes e familiares, auxiliando-os a ultrapassar as barreiras socioeconômicas, culturais, burocráticas e psicológicas que dificultam o acesso os serviços e sistemas de saúde, além de otimizar a adesão e contribuir na efetividade do tratamento^(2)^.

Estudos avaliando a atuação do ONN mostraram que pacientes com câncer de pulmão acompanhados pelo ONN iniciaram seu tratamento mais precoce em relação aos pacientes não acompanhados pelos mesmos^(3,4)^. Outro estudo revelou uma redução no tempo entre o diagnóstico do câncer de mama e o início do tratamento^(5)^. Ambas publicações destacam a relevância da atuação do ONN.

Diante dessas evidências, no Brasil, gradativamente, estão sendo implantados programas de navegação para pacientes oncológicos em diferentes centros de alta complexidade em oncologia. Apesar disso, ainda há poucos estudos mostrando a efetividade do ONN, especialmente voltados aos pacientes cirúrgicos com CCP.

Assim, considerando-se a complexidade do cuidado com o paciente com CCP, torna-se relevante estudar a atuação do ONN na navegação durante o tratamento cirúrgico desses indivíduos.

## OBJETIVOS

Avaliar o impacto da atuação do ONN no tratamento de pacientes cirúrgicos com CCP.

## MÉTODOS

### Aspectos éticos

A pesquisa foi aprovada pelo Comitê de Ética em Pesquisa, em conformidade com a Resolução brasileira n^o^ 466/2012 para pesquisas envolvendo seres humanos. Foi realizada aplicação do Termo de Consentimento Livre e Esclarecido (TCLE) em todos os pacientes que havia disponibilidade e que vieram ao serviço. Para os casos de óbitos, foi solicitada dispensa do TCLE. Para os casos de impossibilidade de ir à instituição, devido à pandemia de COVID-19, necessidade de isolamento social ou outra condição limitante, foi fornecido o consentimento por telefone por meio do *software* Iris Dialtech^®^ (padronizada na instituição). As ligações foram gravadas, garantindo que o paciente entendesse a garantia de confidencialidade, concordando com o uso de seus dados. Este artigo constitui o resultado de dissertação de mestrado intitulada “Atuação do enfermeiro navegador no desfecho dos cuidados pré e pós-operatórios em pacientes com tumor de cabeça e pescoço no Instituto do Câncer do Estado de São Paulo”. A referida dissertação está disponível na Biblioteca Digital de Teses e Dissertações da Universidade de São Paulo, e pode ser acessada por meio do seguinte *link:* https://doi.org/10.11606/D.5.2024.tde-12092024-163018.

### Desenho, período e local do estudo

Trata-se de estudo observacional, tipo coorte retrospectiva, quantitativo, realizado em um Centro de Assistência de Alta Complexidade em Oncologia, que recebe e trata os pacientes com CCP advindos de todas as regiões do Brasil e América Latina, regulado pelo Sistema Único de Saúde, no período de dezembro 2018 a março de 2021.

Neste referido local de estudo, há o programa de atendimento ao paciente com CCP pelo ONN, que acontece de forma sistematizada da seguinte forma:

Os pacientes chegam ao nosso serviço já com o diagnóstico de CCP. Ao passarem em primeira consulta com equipe da cirurgia de cabeça e pescoço, os que são classificados com o estadiamento >II são encaminhados para avaliação com ONN que, por meio da consulta de enfermagem, levantam as demandas de saúde apresentadas pelo paciente (*e.g*., demandas relacionadas aos aspectos sociais, nutricionais, econômicas, psicológicas, entre outras), encaminhando o paciente para atendimento pela equipe multidisciplinar.

O ONN realiza o acompanhamento e gerenciamento dos agendamentos, avalia os motivos das ausências no tratamento, e faz busca ativa, quando necessário, a fim de facilitar a jornada e processos do paciente durante o tratamento. Além disso, o ONN realiza a discussão do caso na reunião clínica, que acontece com participação das equipes interdisciplinares, incluindo as equipes médicas da cirurgia, oncologia, radioterapia e radiologia. Nesta reunião, (chamada de *Tumor Board*) eram analisados os exames de estadiamento, abordando as questões sociais, nutricionais e psicológicas do paciente que poderiam influenciar de alguma forma o tratamento. Nesta discussão clínica, define-se se o paciente seguirá com a programação cirúrgica ou se terá uma mudança de conduta para tratamento clínico. O centro oncológico dispõe de um programa de atendimento por telefone aos pacientes, familiares e cuidadores denominado “Alô, Enfermeiro”, com atendimento 24 horas por dia, pelo qual os pacientes e seus familiares/cuidadores podem esclarecer suas dúvidas referentes ao tratamento e/ou procedimentos sem sair de suas residências, proporcionando uma maior comodidade e segurança.

As recomendações do *STrengthening the Reporting of OBservational studies in Epidemiology* nortearam o desenho do estudo.

### População ou amostra; critérios de inclusão e exclusão

Foram incluídos os pacientes com diagnósticos de CCP, após primeira consulta médica na instituição, em estágios II a IV da doença, que tinham indicação cirúrgica de grande porte e que tiveram indicação de traqueostomia e uso de cateter nasoentereal. Excluíram-se os que tinham tumores malignos da glândula tireoide, glândulas salivares e pele.

### Protocolo do estudo

O estudo foi dividido em duas fases:

- 1º fase (estadiamento): esta fase foi composta pelos pacientes CCP assistidos desde a primeira consulta institucional (PCI) (momento de admissão do paciente ao hospital) até a definição de conduta, após os exames de estadiamento e discussão no *Tumor Board*/retorno médico;
- 2ª fase (operatória): esta fase foi composta após a definição de conduta no *Tumor Board*/retorno médico até o retorno pós-operatório. Nesta fase, foram excluídos pacientes cuja conduta tenha sido apenas o acompanhamento clínico.

O cálculo do tamanho da amostra foi baseado na comparação da atuação do ONN (grupo intervenção - pacientes que foram acompanhados pelo ONN), considerando um erro tipo I de 5% e o poder estatístico de 80%, com a hipótese de que 50% do grupo intervenção teriam o melhor seguimento ao tratamento e a hipótese nula em que 30% do grupo controle (não acompanhados pela ONN, seguiram o fluxo institucional) teriam melhor seguimento ao tratamento.

O grupo controle e intervenção foram divididos em dois subgrupos cada um. Assim, tivemos:

- Grupo intervenção e cirurgia: pacientes que foram acompanhados pelo ONN e realizaram cirurgia;
- Grupo intervenção e tratamento clínico: pacientes que foram acompanhados pelo ONN, sendo indicado tratamento clínico;
- Grupo controle e cirurgia: pacientes que não foram acompanhados pelo ONN e realizaram cirurgia;
- Grupo controle e tratamento clínico: pacientes que não foram acompanhados pelo ONN, sendo indicado tratamento clínico.

O número de casos foi de 40 pacientes no grupo intervenção, sendo que, após a 1ª fase, 25 foram encaminhados para cirurgia (grupo intervenção e cirurgia) e 15 foram encaminhados para conduta clínica (grupo intervenção e tratamento clínico). Já no grupo controle, dos 120, 75 pacientes foram operados (grupo controle e cirurgia), e 45 foram encaminhados à conduta clínica (grupo controle e tratamento clínico), sendo considerado 3:1, como mostra a Figura 1.

**Figura 1:** Descrição dos grupos

A coleta dos dados quantitativos ocorreu por meio de pesquisa retrospectiva de informações em prontuário eletrônico dos pacientes que participaram do acompanhamento do ONN (grupos intervenção e cirurgia, e grupos intervenção e tratamento clínico), comparados com os pacientes que passaram no ambulatório no mesmo período de tempo com as mesmas características (CID 10 - classificação internacional de doenças - estadiamento da doença), sendo separada uma planilha com pacientes que fizeram cirurgia e pacientes que fizeram tratamento clínico. Esta planilha foi inserida no sistema Randon, e os pacientes foram aleatorizados até encontrarmos o n de pacientes para os grupos controle (grupo controle e cirurgia) e intervenção (grupo controle e tratamento clínico).

Para subsidiar a coleta de dados, foram elaborados um instrumento de coleta de dados e uma base de dados na plataforma REDCap^®^ para registrar informações coletadas dos prontuários eletrônicos. Todos os dados coletados foram confidenciais, com acesso somente pelo investigador, assegurando a confidencialidade dos dados.

Para avaliar a atuação e os benefícios do atendimento do ONN aos pacientes com CCP, foram avaliadas as variáveis a seguir e comparadas entre o grupo intervenção e controle: nível de escolaridade; avaliação de performance clínica pelo escore da *Eastern Cooperative Oncologic Group*, Índice de Massa Corporal e Índice de Desenvolvimento Humano (IDH); tempo entre a PCI e realização dos exames de estadiamento; tempo decorrido do diagnóstico ao início do tratamento; tempo entre a primeira consulta nutricional e agendamento com equipe interdisciplinar (nutrição, fonoaudiologia, serviço social, psicologia e odontologia); tempo de internação; número de pacientes que procurou pronto atendimento; número de pacientes que contataram o serviço “Alô, Enfermeiro”.

### Análise dos resultados e estatística

As variáveis qualitativas foram apresentadas na forma de frequência absoluta (n) e relativa (%), e as variáveis quantitativas foram apresentadas na forma de média, mediana, valores mínimos e máximos e desvio padrão. A associação entre as variáveis qualitativas foi analisada com auxílio dos testes qui-quadrado de Pearson ou teste exato de Fisher. Já a comparação entre as variáveis quantitativas entre os grupos foi realizada pelo teste t de Student ou Mann-Whitney, dependendo das distribuições dos dados. O nível de significância adotado foi de 5%. As análises estatísticas foram realizadas com auxílio do *software* estatístico *Statistical Package for the Social Sciences*, versão 18, para *Windows*.

## RESULTADOS

Após atenderem aos critérios de elegibilidade, foram incluídos no estudo total de 160 pacientes, dos quais 40 compuseram o grupo intervenção, e 120 foram incluídos no grupo controle.

Na população que compôs o estudo, a idade variou de 30 a 85 anos entre os homens e mulheres, sendo a mediana de 60,3 no grupo intervenção, e 53,5, no grupo controle.

Quanto ao sexo, tanto no grupo intervenção quanto no controle, observaram-se a predominância do sexo masculino, bem como a maior frequência do ensino fundamental incompleto, IDH alto em ambos os grupos e estadiamento T4.

Em relação ao diagnóstico, a neoplasia de língua foi a mais predominante, tanto no grupo intervenção quanto no grupo controle, não apresentando diferença estatisticamente significativa entre os grupos.

Foi realizado levantamento do intervalo de tempo entre a data da PCI do paciente e a data em que ele foi atendido pela equipe multidisciplinar. Para as equipes de fonoaudiologia, psicologia e serviço social, o grupo intervenção foi atendido em cerca de dois dias, dois dias e 1,37 dias, respectivamente. Isso representa uma redução de aproximadamente 93% em relação ao grupo controle, que aguardou 30 dias, 25,6 dias e 33,64 dias, respectivamente. Para a realização de tomografia, o grupo intervenção aguardou uma média de 9,24 dias, enquanto o grupo controle aguardou 19,77 dias. Isso representa uma redução de aproximadamente 53% no tempo de espera (de 19,77 dias para 9,24 dias). A diferença foi estatisticamente significativa (p = 0,01) No entanto, observa-se que o grupo intervenção levou, em média, mais tempo para passar pela avaliação odontológica em comparação ao grupo controle (Tabela 1).

**Tabela 1:** Descrição dos grupos intervenção e controle segundo o intervalo de tempo da primeira consulta institucional com a equipe interdisciplinar e exames de estadiamento (tomografia e endoscopia - Instituto do Câncer do Estado de São Paulo - Hospital das Clínicas da Faculdade de Medicina da Universidade de São Paulo - 2018 a 2021), São Paulo, São Paulo, Brasil, 2024

	Grupo controle		Grupo intervenção		Valor de *p*
	Média	Desvio padrão	Média	Desvio padrão	
Entre PCI e consulta de enfermagem	15,89	69,66	0,11	0,46	0,004^3^
Entre PCI e consulta de nutrição	18,71	29,77	1,68	0,67	0,113^3^
Entre PCI e consulta de fonoaudiologia	30	24,97	2	<0,01	**0,000^3^**
Entre PCI e consulta de psicologia	25,6	31,96	2	<0,01	**0,000^3^**
Entre PCI e consulta de serviço social	33,64	33,61	1,37	0,96	**0,000^3^**
Entre PCI e consulta de dentista	33,23	34,14	44,28	172,66	**0,000^3^**
Entre PCI e tomografia	17,56	23,03	6,72	5,9	**0,001^3^**
Entre PCI e endoscopia	19,77	23,94	9,24	5,48	0,321^3^

3 Teste Mann-Whitney; PCI - primeira consulta institucional.

Em relação à adesão ao tratamento, verificou-se que o grupo intervenção apresentou melhor aderência ao tratamento quando comparado ao grupo controle, apresentando associação estatística significante (p<0,001) (Tabela 2).

**Tabela 2:** Descrição dos grupos intervenção e controle segundo as avaliações referentes à adesão dos pacientes em consultas (Instituto do Câncer do Estado de São Paulo - Hospital das Clínicas da Faculdade de Medicina da Universidade de São Paulo - 2018 a 2021), São Paulo, São Paulo, Brasil, 2024

	Grupo controle		Grupo intervenção		Valor de *p*
	n	%	n	%	
Sim	51	42,5	30	75	**<0,001^2^**
Não	69	57,5	10	25	
Faltas	69	57,5	10	25	
Sem acompanhante^*^	NA	NA	2	5	
Sem condição financeira^*^	NA	NA	3	7,5	
Esquecimento^*^	NA	NA	<0,01	<0,01	

^2^Teste exato de Fisher;

* Grupo intervenção e grupo controle; NA - não aplicável.

Em relação ao tempo entre a primeira consulta e a cirurgia, a média em dia foi menor no grupo intervenção. O tempo de internação (entre a cirurgia e a alta) foi maior no grupo intervenção, apesar de não haver associação estatística significativa entre os grupos. Este resultado pode ser evidenciado na Tabela 3.

**Tabela 3:** Descrição do grupo intervenção e cirurgia e do grupo controle e cirurgia segundo o intervalo de tempo entre a primeira consulta institucional e a cirurgia, e a cirurgia e a alta (Instituto do Câncer do Estado de São Paulo - Hospital das Clínicas da Faculdade de Medicina da Universidade de São Paulo - 2018 a 2021), São Paulo, São Paulo, Brasil, 2024

		Grupo controle e cirurgia	Grupo intervenção e cirurgia	Valor de *p*
**Tempo entre 1º consulta e cirurgia (dias)**	Média	65,75	41,92	0,006^3^
	Mediana	54	34	
	Mínimo	13	19	
	Máximo	301	89	
**Tempo desde cirurgia até a alta (dias)**	Média	17,39	28,48	0,4393^3^
	Mediana	8	10	
	Mínimo	0	2	
	Máximo	360	356	

*^3^Teste Mann-Whitney.*

Em relação à procura pelo serviço do “Alô, Enfermeiro”, verificou-se que foi maior no grupo controle e cirurgia quando comparado aos outros grupos.

As dúvidas sobre os cuidados com o cateter nasoenteral e a traqueostomia foram as mais frequentes entre os pacientes dos grupos controle e intervenção submetidos à cirurgia. Já entre aqueles em tratamento clínico, a principal queixa foi a dor não paliada. No entanto, não houve diferença estatisticamente significativa entre os grupos (Tabela 4).

**Tabela 4:** Descrição dos grupos intervenção (tratamento clínico e cirurgia) e controle (tratamento clínico e cirurgia) segundo procura pelo “Alô, Enfermeiro” e os motivos da ligação, São Paulo, São Paulo, Brasil, 2024

	Grupo controle e cirurgia		Grupo intervenção e cirurgia		Valor de *p*	Grupo controle e tratamento clínico		Grupo intervenção e tratamento clínico		Valor de *p*
	**n**	**%**	**n**	**%**		**n**	**%**	**n**	**%**	
**Ligação “Alô, Enfermeiro”**					0,107^2^					0,541^2^
Sim	42	56,00	9	36,00		17	37,80	4	26,70	
Não	33	44,00	16	64,00		28	62,30	11	73,30	
**Motivo**										
Dúvida relacionada aos cuidados (cateter nasoenteral/traqueostomia)	16	38,10	2	22,20		1	5,90	<0,01	<0,01	
Dor	3	7,10	<0,01	<0,01		7	41,20	1	25,00	
Queixas clínicas (hipotensão/mal-estar/secreção ferida operatória/febre)	16	38,10	2	22,20		4	23,50	<0,01	<0,01	
Dúvidas sobre agendamentos	11	26,20	4	44,40		3	17,60	3	75,00	
Contato com navegador	<0,01	<0,01	1	11,10		<0,01	<0,01	<0,01	<0,01	
Outros	5	11,90	2	22,20		1	5,90	<0,01	<0,01	

2 Teste exato de Fisher. O tempo de acompanhamento e ligações no “Alô, Enfermeiro” foi da primeira consulta institucional até a consulta de pós-operatório para o grupo intervenção/controle e cirurgia, e da primeira consulta institucional até antes de iniciar o tratamento clínico para o grupo intervenção/controle e tratamento clínico.

Em relação à ida ao pronto atendimento, observa-se que a procura foi maior no grupo controle e cirurgia, como também pelo grupo controle e tratamento clínico. A queixa mais frequente em ambos os grupos foi a dor não paliada, apresentando diferença estatisticamente significante de p<0,001 e p=0,003,respectivamente, como revelou a Tabela 5.

**Tabela 5:** Descrição dos grupos intervenção (tratamento clínico e cirurgia) e controle (tratamento clínico e cirurgia) segundo comparecimento ao pronto atendimento e seu motivo, São Paulo, São Paulo, Brasil, 2024

	Grupo controle e cirurgia		Grupo intervenção e cirurgia		Valor de *p*	Grupo controle e tratamento clínico		Grupo intervenção e tratamento clínico		Valor de *p*
	**n**	**%**	**n**	**%**		**n**	**%**	**n**	**%**	
**Ida ao pronto atendimento**					0,000^2^					0,003^2^
Sim	61	81,3	7	28,0		35	77,8	5	33,3	
Não	14	18,7	18	72,0		10	22,2	10	66,7	
**Motivo**										
Dor	24	40,0	<0,01	<0,01		10	28,6	2	40,0	
Sangramento	3	5,6	<0,01	<0,01		2	5,7	1	20,0	
Dispneia	2	3,1	1	14,2		6	17,1	1	20,0	
Perda de cateter nasoenteral	14	23,3	1	14,2		3	8,6	1	20,0	
Relacionado à traqueostomia	1	1,7	<0,01	<0,01		7	20,4	<0,01	<0,01	
Outras infecções	7	11,7	2	28,5		<0,01	<0,01	<0,01	<0,01	
Feridas operatórias	6	8,1	1	14,2		4	11,4	<0,01	<0,01	
Perda de dreno	1	1,7	<0,01	<0,01		<0,01	<0,01	<0,01	<0,01	
Outras	3	5,6	2	28,5		3	8,6	<0,01	<0,01	

2 Teste exato de Fisher. O tempo de acompanhamento e ligações no “Alô, Enfermeiro” foi da primeira consulta institucional até a consulta de pós-operatório para o grupo intervenção/controle e cirurgia, e da primeira consulta institucional até antes de iniciar o tratamento clínico para o grupo intervenção/controle e tratamento clínico.

## DISCUSSÃO

Este estudo indicou que o ONN demonstra ter impacto sobre o atendimento perioperatório de pacientes com CCP. A vantagem deste estudo é a análise simultânea de dois grupos com CCP acompanhados em um centro terciário de oncologia. Outro ponto forte é a abordagem do ONN, uma vez que esse profissional integrou e coordenou uma equipe multidisciplinar (nutrição, fonoaudiologia, serviço social, psicologia e odontologia).

O perfil dos pacientes com CCP estudado foi predominantemente do sexo masculino, 94 (78,3%) nos grupos controle e 35 (87,5%) nos grupos intervenção. O nível de escolaridade da população estudada foi de 75 (62,5%) nos grupos controle e 27 (67,5%) nos grupos intervenção com ensino fundamental incompleto. O gênero masculino, assim como baixa escolaridade, é predominante em diversos artigos quando se fala em CCP^(6-9)^.

O estadiamento avançado T4 foi predominante em ambos os grupos, indicando que os pacientes já chegaram na instituição com o tumor localmente avançado, o que, em termos de tratamento, significa uma abordagem mais agressiva, mais custosa e com menor qualidade de vida ao paciente. Tumores com estadiamento T4 estão associados à sobrevida reduzida, principalmente em CCP^(10,11)^. Devido à maior incidência de pacientes diagnosticados em estadiamento 4, é fundamental que esses indivíduos recebam um acompanhamento rigoroso e contínuo. Isso assegura que o tratamento seja iniciado o mais rapidamente possível, maximizando as chances de eficácia e melhorando os resultados clínicos.

Visando à necessidade do início do tratamento precoce, este estudo levantou as variáveis de tempo, que já são confirmadas em evidências prévias na literatura, que o ONN otimiza o início do tratamento em pacientes com câncer^(5,12-15)^. O presente estudo comparou o tempo de atendimento com a equipe interdisciplinar e exames de estadiamento entre os grupos. Verificou-se que o tempo médio que o grupo intervenção passou com as equipes de fonoaudiologia foi de dois dias, e o grupo controle (ganho de 28 dias), 30 dias; para a psicologia: dois dias para o grupo intervenção e 28,5 dias para o grupo controle (diminuição de 26 dias); serviço social: 1,37 dias no grupo intervenção e 33,5 dias no grupo controle (redução de 31 dias). Já o exame de tomografia foi realizado em uma média de 6,72 dias pelo grupo intervenção e 17,5, pelo grupo controle (dez dias a menos), apresentando associação estatística significante (p<0,001) na população estudada.

Outro resultado relevante está relacionado à adesão ao tratamento oncológico. Segundo a Organização Mundial da Saúde, a adesão é um fenômeno multidimensional influenciado por cinco conjuntos de fatores: socioeconômicos (pobreza, analfabetismos, etc.); da equipe de saúde e relacionados ao sistema (serviços de saúde pouco desenvolvido consultas curtas, etc.); relacionados à condição (sintomas relacionados à progressão da doença, disponibilidade de tratamentos eficazes); relacionados à terapia; e relacionados ao paciente (esquecimento, estresse psicossocial, ansiedade, entre outros)^(16)^.

Assim, a adesão do paciente é crucial para o sucesso do tratamento, mostrando-se desafiadora, uma vez que envolve aspectos comportamentais e subjetivos. O método escolhido no presente estudo para mensuração da adesão foi o levantamento dos dados por meio dos registros das consultas médicas, equipe interdisciplinar e exames. Os pacientes que tinham duas ou mais faltas não justificadas em consulta/exames eram considerados com má adesão.

A partir desta definição, o presente estudo encontrou no grupo intervenção que foram acompanhados pelo ONN uma adesão em 30 (75%) das consultas e exames agendados, comparado com 51 (42,5%) no grupo controle (p<0,01). Os estudos trazem que os pacientes acompanhados pelo ONN apresentam melhor adesão ao tratamento. Isso se dá por fatores como uma melhora satisfação do paciente, que reflete uma maior adesão^(17-19)^.

Outro ponto importante é o vínculo criado entre o paciente e o ONN. Ao realizar o atendimento, é importante proporcionar um ambiente acolhedor, mostrando respeito e empatia naquele momento, além de realizar adaptação cultural, respeitando as crenças e valores do paciente a partir de uma comunicação efetiva, pela qual é criado um laço de confiança por parte do paciente com aquele enfermeiro. Esse vínculo vai se fortalecendo a cada consulta e ligação que é realizada, pela qual o paciente se sente mais confortável para falar sobre suas dificuldades, incluindo se haverá algum impedimento à sua ida à instituição por falta de condição financeira, por exemplo, dando oportunidade para que seja acionado o serviço social e, assim, consiga-se evitar a falta deste paciente na consulta, destacando-se, assim, a interface do e ONN com o serviço social.

Especificamente sobre a influência do ONN sobre os grupos intervenção e controle cirúrgico, é importante destacar que o tempo entre a primeira consulta e a cirurgia foi menor para o grupo intervenção e cirurgia, com uma mediana de 34 dias e 54 dias para o grupo controle e cirurgia. A agenda cirúrgica permaneceu inalterada, não havendo modificações na sequência das operações. Os pacientes do grupo intervenção e cirurgia foram submetidos à cirurgia, conforme programado, sem alterações na ordem de prioridade; essa discrepância no tempo pode ser atribuída à melhoria na aderência ao tratamento por parte dos pacientes do grupo intervenção.

Quando surgiam ausências em exames ou consultas nesses grupos, o ONN realizava uma busca ativa para localizar esses pacientes, remarcando os exames/consultas, entendendo o motivo da falta e criando um plano de ação para o auxílio desse paciente. Como resultado, eles ficavam prontos para a cirurgia em um intervalo de tempo mais curto, contribuindo para a diferença observada. Destaca-se aqui que esse tipo de paciente necessita de um monitoramento mais de perto, por todos os aspectos já citados anteriormente.

Em relação ao tempo de internação desde a cirurgia até a alta, não se obteve uma significância estatística entre os grupos intervenção e controle, porém a mediana foi de dez dias, para o grupo intervenção e cirurgia, e oito dias, para o grupo controle e cirurgia. Na literatura, encontrou-se um resultado diferente deste, sendo que estudo investigou a eficácia das intervenções sistematicamente planejadas pelo ONN em pacientes recentemente diagnosticados com câncer em geral. Os resultados indicaram que os pacientes que receberam assistência do ONN apresentaram um período de internação hospitalar menor, em comparação ao grupo controle e cirurgia^(19-21)^.

Apesar de ser uma diferença pequena no resultado (dois dias), uma possível justificativa para a extensão do tempo no grupo intervenção e cirurgia foi que alguns pacientes do grupo intervenção tinham baixo suporte familiar, necessitando de um maior suporte social para o planejamento da alta, o que aumentou o tempo de internação.

Mesmo considerando o conhecimento da equipe interdisciplinar, alguns desafios surgiram no período pós-operatório, pois esses pacientes precisaram ser encaminhados a uma instituição de apoio capaz de proporcionar cuidados integrais. No entanto, a obtenção de uma vaga neste tipo de instituição só era possível quando o paciente estava em condições de alta e quando havia a disponibilidade de uma vaga. Essa espera, em algumas ocasiões, resultou em uma demora considerável, contribuindo para a elevação da mediana no tempo (em dias) de internação. Identificou-se, portanto, a necessidade de um aprimoramento do sistema de referência e contrarreferência do Sistema Único de Saúde, reconhecendo a importância de um esforço coordenado para otimizar a transferência dos pacientes com demandas específicas, como é o caso dos pacientes com CCP em recuperação pós-operatória.

A literatura é clara em destacar que a atuação de ONN tem um impacto positivo na redução das visitas ao pronto atendimento^(21,22)^. Ao analisar-se a incidência dos pacientes que procuraram o pronto atendimento neste estudo, observou-se que 61 (81,3%) do grupo controle e cirurgia que passaram por cirurgia necessitaram de consultas no pronto atendimento, em comparação com sete (28%) do grupo intervenção e cirurgia. No caso do tratamento clínico, 35 (77,8%) do grupo controle e tratamento clínico procuraram o pronto atendimento, em comparação com cinco (33,3%) do grupo intervenção e tratamento clínico (p<0,01).

Uma análise dos motivos que levaram esses pacientes ao pronto atendimento revelou a dor como a principal causa em todos os grupos do estudo, mesmo após as orientações frequentes aos pacientes sobre o uso adequado de medicações. Durante as consultas do ONN, bem como nas discussões com a equipe médica, a dor era sempre discutida, bem como as necessidades de ajuste medicamentoso.

Uma das estratégias de navegação é a telenfermagem, definida pelo *International Council of Nurses* como a prática de enfermagem à distância por meio eletrônico que tem apresentado benefícios significativos, sendo reconhecida como uma ferramenta para o manejo de toxicidades e sintomas em pacientes oncológicos^(23)^.

No referido centro oncológico, Instituto do Câncer do Estado de São Paulo, há o programa denominado “Alô, Enfermeiro”, que consiste no atendimento e monitoramento telefônico do enfermeiro aos pacientes em tratamento oncológico, cirúrgico e clínico. O número de atendimentos diários tem aumentado progressivamente ao longo dos anos na instituição. Em 2021, o programa realizou mais de 54 mil atendimentos receptivos, nos quais o paciente ou acompanhante entra em contato com a central do programa, com cerca de 30 mil atendimentos ativos, nos quais o enfermeiro entra em contato com o paciente ou acompanhante^(23)^.

O presente estudo avaliou as ligações dos pacientes acompanhados pelo ONN para o “Alô, Enfermeiro”. Os resultados obtidos mostram 42 (56,0%) do grupo controle e cirurgia e 17 (37,8%) do grupo controle e tratamento clínico, comparados com nove (36,0%) do grupo intervenção e cirurgia e quatro (26,7%) do grupo intervenção e tratamento clínico. Os principais motivos das ligações dos pacientes/familiares foram as dúvidas relacionadas aos cuidados, como cuidados com cateter nasoenteral e traqueostomia em ambos os grupos. Destaca-se, também, a maior procura (7; 41,2%) no grupo controle e tratamento clínico por queixa de dor não paliada, levantando, assim, a necessidade de uma melhor cobertura de analgesia nos pacientes com CCP que estão em tratamento clínico. A atuação de ONN tem sido amplamente reconhecida como uma estratégia eficaz para reduzir a necessidade de buscas por atendimento médico, incluindo visitas ao pronto-socorro e chamadas telefônicas^(19,20,22)^.

Sabe-se que a atuação do ONN consiste em ser um facilitador para o paciente e família acerca das ações durante todo o processo do tratamento oncológico. Além disso, eles contribuem para a diminuição do tempo para a realização dos procedimentos, redução das idas ao pronto atendimento, melhor esclarecimento das dúvidas que vão surgindo em cada fase do tratamento, bem como ser um porto seguro para o paciente e sua família, pois eles sabem que têm um profissional de referência que os ajuda a coordenar tudo o que necessita ser realizado nesta fase de sua vida.

Em relação ao aspecto gerencial, a efetividade das ações do ONN se reflete na possibilidade de elaborar indicadores, como redução do tempo para realização de exames de estadiamento, avaliações pela equipe multidisciplinar, tempo desde a PCI até a cirurgia, tempo de internação, além do monitoramento da adesão ao tratamento, ligação no “Alô, Enfermeiro” e idas ao pronto atendimento.

### Limitações do estudo

Como limitação desta pesquisa, destaca-se a diminuição do número de pacientes atendidos pelo ONN durante a pandemia de COVID-19, visto que os pacientes não eram encaminhados para PCI devido às restrições sanitárias; por isso, há um número reduzido de pacientes do grupo intervenção.

### Contribuições para a área da saúde, enfermagem ou políticas públicas

Este estudo contribui de forma significativa para o campo da enfermagem ao evidenciar os benefícios da atuação do ONN, assim como mostrou resultados positivos nos indicadores de tempo, adesão e diminuição de idas ao pronto atendimento. Quando considerada a redução das visitas dos pacientes ao pronto atendimento, pondera-se sobre a diminuição dos custos associados. Embora o presente estudo não tenha abordado diretamente a redução de custos, estudos futuros podem abordar essa temática.

## CONCLUSÕES

Os resultados deste estudo permitiram avaliar o impacto assistencial positivo com a implantação do ONN tanto para os pacientes com CCP quanto para a instituição.

Quando comparados o grupo intervenção com o grupo controle, pode-se concluir que, para os pacientes cirúrgicos com CCP, foi possível constatar que o ONN conseguiu melhorar a adesão ao tratamento, bem como reduziu o tempo para exames, consultas com equipe multidisciplinar, assim como a diminuição de uma mediana de 20 dias para a cirurgia. Também, foi observado que os pacientes navegados pelo ONN tiveram menos idas ao pronto atendimento e menos ligações ao “Alô, Enfermeiro”.

Assim, este estudo permitiu confirmar que a atuação do ONN como coordenador de cuidado fazendo a interface com a equipe interdisciplinar propicia uma comunicação mais efetiva, caracterizando-se como um elo entre a equipe assistencial, a fim de atender às necessidades de saúde biopsicossociais apresentadas pelos pacientes com CCP.

Por fim, o ONN coloca o paciente no centro do cuidado e, assim, torna-se uma referência tanto para o paciente e seu familiar, na difícil jornada do tratamento oncológico, quanto para a equipe assistencial, na coordenação das ações assistenciais necessárias a cada paciente, bem como para a instituição, ao contribuir com a identificação de sinais precoces de complicações e com a redução de dias de internação.

## Data Availability

Os dados de pesquisa só estão disponíveis mediante solicitação.
